# Gene expression profiles in rat brain disclose CNS signature genes and regional patterns of functional specialisation

**DOI:** 10.1186/1471-2164-8-94

**Published:** 2007-04-04

**Authors:** Christine Stansberg, Audun Osland Vik-Mo, Rita Holdhus, Harald Breilid, Boleslaw Srebro, Kjell Petersen, Hugo A Jørgensen, Inge Jonassen, Vidar M Steen

**Affiliations:** 1 Dr E. Martens Research Group for Biological Psychiatry and Bergen Mental Health Research Center, Department of Clinical Medicine, University of Bergen, Norway; 2 Center for Medical Genetics and Molecular Medicine, Haukeland University Hospital, Norway; 3 Department of Biomedicine, University of Bergen, Norway; 4 Department of Informatics, University of Bergen, Norway; 5 Computational Biology Unit, Bergen Centre for Computational Science, University of Bergen, Norway; 6 Section for Psychiatry, Department of Clinical Medicine, University of Bergen, Norway

## Abstract

**Background:**

The mammalian brain is divided into distinct regions with structural and neurophysiological differences. As a result, gene expression is likely to vary between regions in relation to their cellular composition and neuronal function. In order to improve our knowledge and understanding of regional patterns of gene expression in the CNS, we have generated a global map of gene expression in selected regions of the adult rat brain (frontomedial-, temporal- and occipital cortex, hippocampus, striatum and cerebellum; both right and left sides) as well as in three major non-neural tissues (spleen, liver and kidney) using the Applied Biosystems Rat Genome Survey Microarray.

**Results:**

By unsupervised hierarchical clustering, we found that the transcriptome within a region was highly conserved among individual rats and that there were no systematic differences between the two hemispheres (right versus left side). Further, we identified distinct sets of genes showing significant regional enrichment. Functional annotation of each of these gene sets clearly reflected several important physiological features of the region in question, including synaptic transmission within the cortex, neurogenesis in hippocampus and G-protein-mediated signalling in striatum. In addition, we were able to reveal potentially new regional features, such as mRNA transcription- and neurogenesis-annotated activities in cerebellum and differential use of glutamate signalling between regions. Finally, we determined a set of 'CNS-signature' genes that uncover characteristics of several common neuronal processes in the CNS, with marked over-representation of specific features of synaptic transmission, ion transport and cell communication, as well as numerous novel unclassified genes.

**Conclusion:**

We have generated a global map of gene expression in the rat brain and used this to determine functional processes and pathways that have a regional preference or ubiquitous distribution within the CNS, respectively. The existence of shared specialised neuronal activities in CNS is interesting in a context of potential functional redundancy, and future studies should further explore the overall characteristics of CNS-specific versus region-specific gene profiles in the brain.

## Background

The mammalian brain is divided into distinct regions with structural and functional similarities and differences. Based on information from decades of neuroanatomical-, neurophysiological- and neurochemical studies, in combination with more recent brain imaging findings, the huge complexity of the CNS has become increasingly evident. The subtypes of neurons, regional cytoarchitecture, variation in neurotransmitter distribution and patterns of regional neuronal communication are major factors to determine the functional specialisation of a certain region, but numerous other variables may also contribute. It is therefore obvious that new and multidisciplinary approaches are needed to further improve our understanding of the mammalian brain.

Microarray-based global gene expression profiling constitutes a valuable research tool, since patterns of gene expression are likely to vary between brain regions in relation to their respective functions. Correlations between the transcriptome in a tissue or organ and its respective physiological functions have been reported, although these studies have been limited to viewing the brain as one organ [1,2]. Lately, several studies have identified sets of genes showing differential expression among various areas of the mammalian brain [3-10], including attempts to associate regional gene expression to anatomical structure and functional activity in the brain [5,7]. Transcriptional profiles of adult mouse brain regions actually carry embryonic 'imprints' depending on the developmental origin of the regions, gene expression in the cerebral cortex is thereby more related to that of other forebrain structures, such as the amygdala and hippocampus, than to midbrain or hindbrain structures [10]. Consequently, region specific genes seem to be collectively involved in biological processes such as development, morphogenesis, and pattern specification [10]. It is interesting to note that four gene expression factors have been found useful for distinguishing between brain regions; these being the regional myelin/oligodendrocyte levels, the resident neuron neurotransmitter type, the neurotransmitter innervation profiles and Ca^2+-^dependent signalling and second messenger systems [3]. In line with this, expression of genes involved in signal transduction and neurogenesis appear to differ more among human and chimpanzee brain regions than other groups of genes [4].

On this background, we aimed at further improving the understanding of some specialised brain regions, based on analysis of patterns of global gene expression in the mammalian brain. The laboratory rat is a frequently used animal model in neuroscience, especially due to long-standing traditions in neurophysiology and cognitive research. In our studies we have used the Applied Biosystems Array Expression system with chemiluminescent-based signal detection. The AB Rat Genome Survey arrays represent 27,088 rat genes, of which more than 12,000 until recently were unique to the Celera database and are at this point exclusively represented on this platform. At least fifty percent of all Celera genes carry functional annotation provided by the proprietary Panther database [11]. In comparison, only about fifteen percent of the public rat genes carry GO-annotation. Our study is thus the largest to date in terms of the number of genes studied as well as the mapped functional annotation.

As a result, we have generated a map of differentially expressed genes in selected regions of the rat brain. We have further functionally classified the subsets of region-enriched genes and demonstrate that the annotations reflect important hallmarks of physiological features of the different brain regions. Finally, we have compared the expression patterns in the brain regions versus some major non-neural tissues and thereby identified a selection of 'CNS signature' genes that reveal characteristics of neuronal processes shared among CNS regions.

## Results

### Quantitative gene expression in different rat tissues

Global gene expression profiles were determined for six different brain regions (fronto-medial-, temporal- and occipital-cortex, hippocampus, striatum and cerebellum; both left and right hemisphere) and three non-CNS tissues (liver, spleen and kidney) in three individual rats. Each brain region was thus in general represented by six replicates; whereas each non-CNS tissue was represented by three replicates. One striatum and one kidney sample did not meet the quality criteria after hybridisation and were thus excluded from the study.

On average, 13,030 genes (48.5%) of the 27,088 genes represented on the microarray displayed reliable hybridisation signal in the 43 samples included in this study (S/N ≥ 3) (Table 1). This number varied between CNS- and non-CNS samples; the average number of genes detected in CNS regions was 14,408 (53.6%), whereas 11,389 (42.4%) were detected in non-CNS tissues.

### Global gene expression profiles of rat brain regions

**Figure 1:**
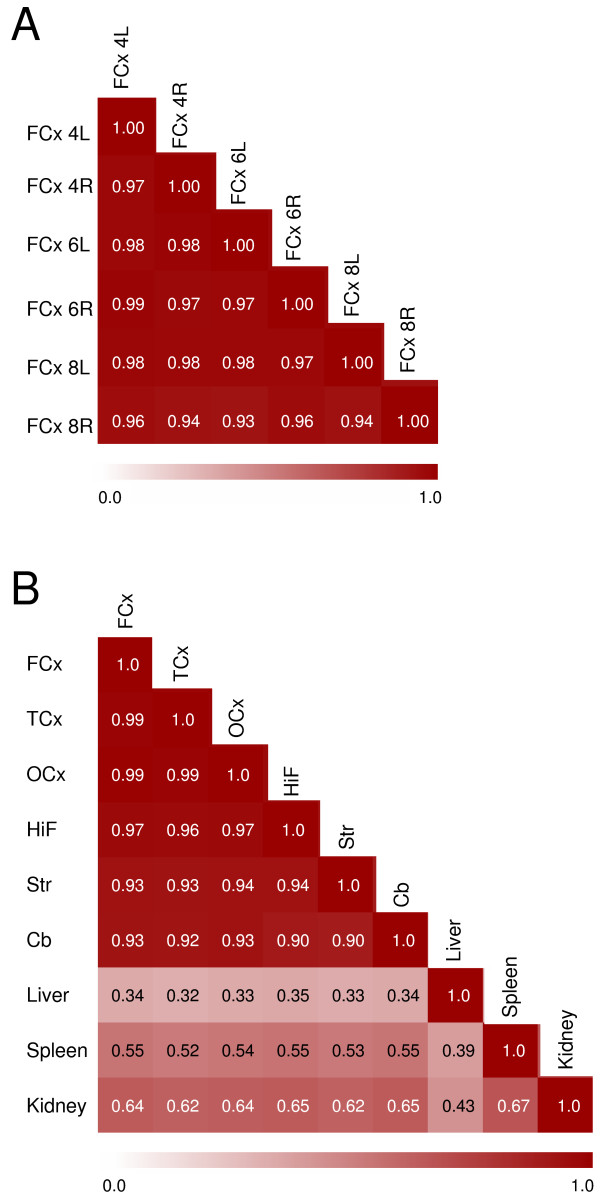
*Correlations of rat tissue transcriptomes*. a) Heat-map illustrating correlations between the six different samples from frontomedial cortex (left and right hemispheres) from the three different rats. b) Heat-map illustrating correlations in average gene expression across tissues and brain regions, all visualised by the Pearson correlation coefficient. Calculations were based on average signal intensities within each non-CNS tissue (3×) and brain region (6×). Red colour indicates the strength of the correlation, ranging from 0.0 (white) to 1.0 (dark red). Numerals (4,6,8) refer to the individual rats. L, left hemisphere; R, right hemisphere. FCx, fronto-medial cortex; TCx, temporal cortex; OCx, occipital cortex; HiF, Hippocampus; Str, Striatum; Cb, cerebellum.

In order to examine interrelationships of global gene expression profiles among the six brain regions and the three non-CNS tissues, Pearson correlation coefficients were calculated. Correlations of all biological replicates within a single region were quite high, from r ~ 0.90 among liver samples to about r ~ 0.99 among cortical samples. The high similarity in global gene expression among cortical samples is exemplified in Fig. 1a by the six samples from frontomedial cortex, showing a heat map of internal correlations of each sample versus every other sample. Furthermore, expression profiles of the various brain regions were quite similar. Using average regional signal intensities, correlations ranged from r = 0.90 between hippocampus and cerebellum to r = 0.99 among the three cortical regions (Fig. 1b). In comparison, correlations were around r = 0.35 for liver compared to any brain region (Fig. 1b)

**Figure 2:**
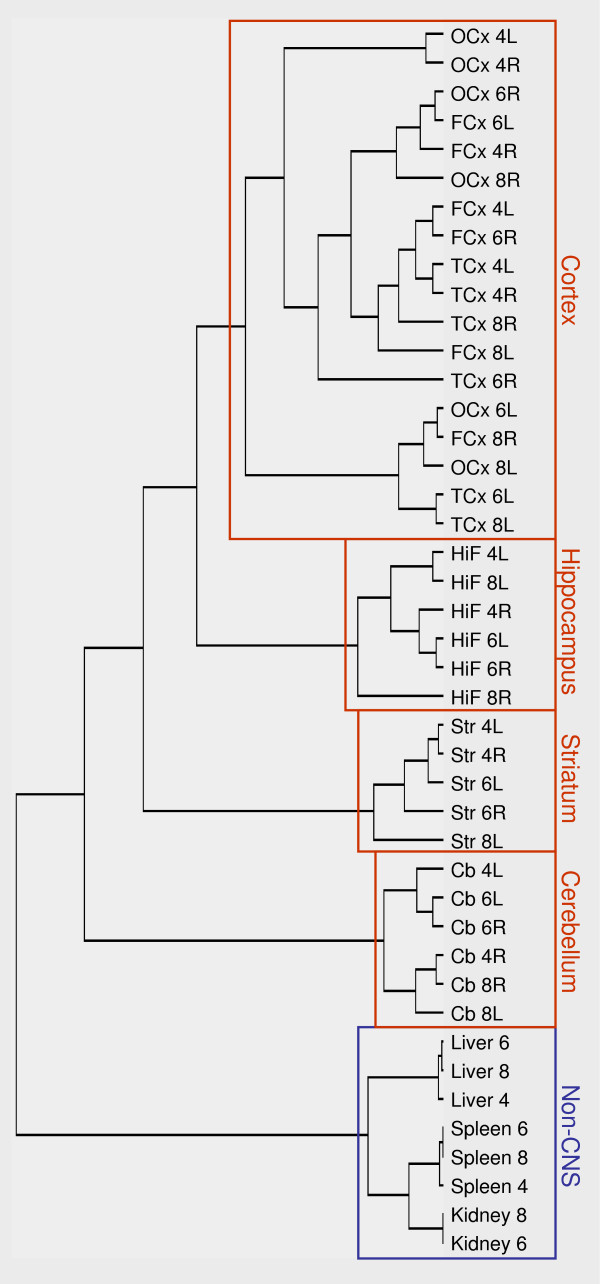
*Hierarchical clustering of rat tissues*. Unsupervised hierarchical clustering showing correlations of expression profiles of all brain and non-CNS rat samples included in this study. The analysis completely separated CNS-regions from non-CNS tissues (blue box), as well as placing all CNS-samples in correct regional branches (red boxes). Numerals (4,6,8) refer to the individual rats. L, left hemisphere; R, right hemisphere. FCx, fronto-medial cortex; TCx, temporal cortex; OCx, occipital cortex; HiF, Hippocampus; Str, Striatum; Cb, cerebellum.

Relationships between the expression profiles of all brain regions and non-CNS tissues were further explored by unsupervised hierarchical clustering. The resulting dendrogram showed two major branches, completely separating CNS samples from non-CNS samples (Fig. 2). The CNS branch had three main internal branching points, perfectly separating the cortical, hippocampal, striatal and cerebellar samples into four different clusters. The transcriptomes of the cortical and the hippocampal samples were most similar, followed by the striatal samples, with the cerebellar samples clearly being the most distinct. The observed relationships between these four different brain structures were robust and not significantly influenced by re-sampling.

Gene expression patterns within a given brain region were highly conserved among individual rats. As shown in Figs. 1a and 2, samples originating from the left and right hemispheres of a certain region from the same rat brain were no closer related to each other than to corresponding samples from the other brains. In addition, no systematic difference between samples from left and right hemispheres could be observed. In accordance with the high correlations (see above), the cortical samples showed no tendency whatsoever to group together according to the sub-region from which they derived, further indicating a very high degree of similarity of sub-cortical transcriptomes among individual rats. Consequently, re-sampling of the dendrogram in Fig. 2 showed that the internal clustering of cortical samples was quite unstable, with most branching points lacking significant bootstrap support (data not shown).

### Genes with regionally enhanced expression patterns

**Figure 3:**
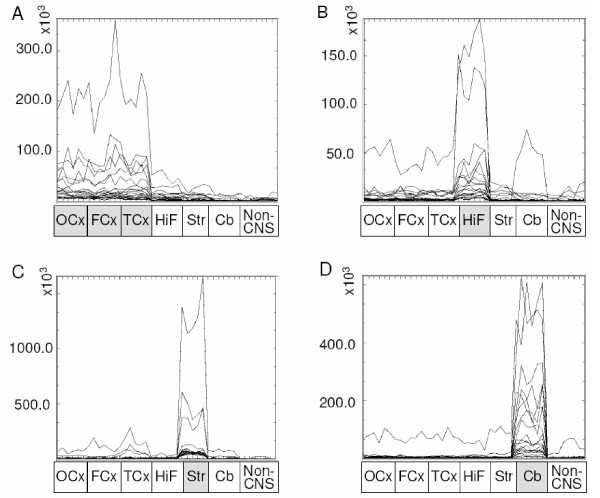
*Regionally enriched expression in rat brain*. Profiles of the 10 top-scoring genes showing enhanced expression in each of the four brain regions; a) cortex, b) hippocampus, c) striatum and d) cerebellum. The 43 individual tissue samples are placed along the x-axis. The y-axis indicates quantile-normalised signal intensities for each gene in each individual sample. FCx, fronto-medial cortex; TCx, temporal cortex; OCx, occipital cortex; HiF, Hippocampus; Str, Striatum; Cb, cerebellum.

The complete subdivision of samples originating from different regions of the brain by hierarchical clustering suggests significant differences in gene expression patterns between these brain regions. Genes showing specific enrichment in one of the four main brain regions; cerebral cortex, hippocampus, striatum or cerebellum; were identified by significance analysis of microarrays (SAM) using a false discovery rate (FDR) of zero. As a result, 353, 189, 314 and 627 genes were found to be significantly enriched in the cortex, hippocampus, striatum and cerebellum, respectively. Intensity profiles of top scoring genes within each region are illustrated in Fig. 3, while the top ten genes for each region are listed in Table 2. The entire list of significantly enriched genes is provided as Additional file 1.

Cerebellum confirmed its diverging profile as shown above by the unsupervised hierarchical clustering and displayed by far the highest number of specifically enriched genes. Interestingly, a large proportion of the genes enriched in striatum or cerebellum apparently showed a more pronounced regional restriction than the genes enriched in cortex or hippocampus (Fig. 3). The latter displayed more balanced patterns of expression, with high levels in the region in question but also some basal expression in other CNS regions, especially hippocampus or cortex, respectively. In line with this, the lowest number of regionally enriched genes was found among the hippocampal samples. As demonstrated in Figs. 1 and 2, hippocampus was most closely correlated to the cortex. Thus, many hippocampal genes probably also have important functions within the cortex.

As described earlier, the hierarchical clustering indicated that gene expression patterns within cortical sub-regions are highly similar, with no systematic differences (Fig. 2). SAM was therefore performed to identify genes with possible enrichment in each of the three sub-regions of the cortex. With a false discovery rate of zero, 43, 64 and 12 genes were found to be significantly enriched in the fronto-medial, temporal and occipital cortex, respectively (data not shown).

For verification purposes, a considerable number of the annotated, regionally enriched genes were either confirmed by the literature or by browsing gene expression databases like the GNF Symatlas v1.1.1 [12-14] and the Gene Expression Omnibus [15]. Validation results for the ten top-scoring genes of each brain region are presented in Table 2, demonstrating a high degree of concurrence with other species and technology platforms. We are therefore of the opinion that additional verification by QPCR is not necessary at this point, since we are not discussing impact of individual genes. Recent platform comparisons have in addition shown high concordances between measurements obtained from commercial microarray platforms and quantitative real time PCR [16,17].

### Functional characterisation of regionally enriched gene sets

**Figure 4:**
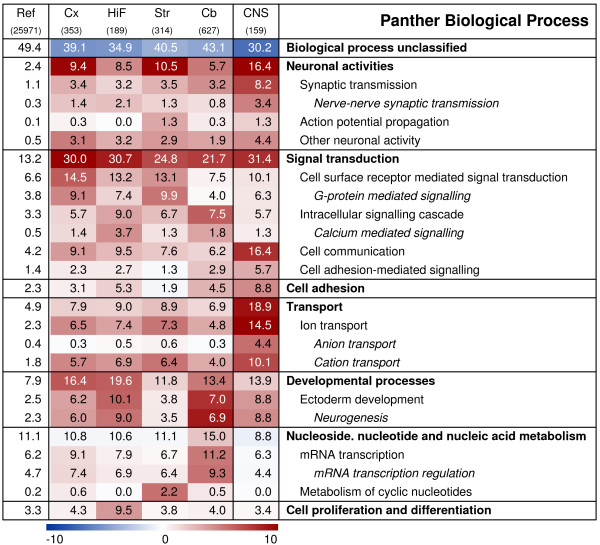
*Functional characterisation of regionally enriched gene sets and CNS-signature genes: Biological Process*. Heat-map demonstrating the significance of over- and under-represented Panther biological processes. Red colour signifies an over-representation of genes mapping to a certain term, blue colour an under-representation and white a representation as expected, based on the overall distribution on the array. The colour intensity indicates the negative log of the significance p-value. Numbers presented on the heat map display the percentage of genes within a gene set that map to a certain term, e.g. 29% of the 353 cortex genes map to the biological process 'signal transduction'. The first column states the overall distribution of a term among the 25,971 genes with detectable expression in the data set, followed by each regional gene set and finally the 'CNS-signature' gene set. FCx, fronto-medial cortex; TCx, temporal cortex; OCx, occipital cortex; HiF, Hippocampus; Str, Striatum; Cb, cerebellum; CNS, 'CNS-signature' genes.

**Figure 5:**
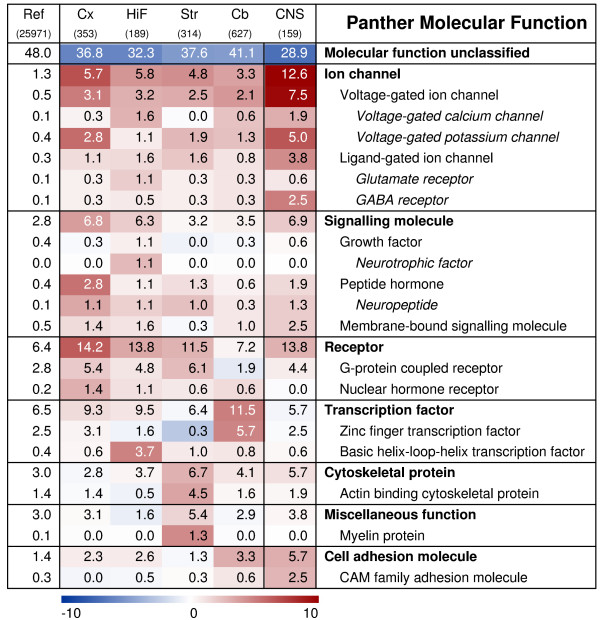
*Functional characterisation of regionally enriched gene sets and CNS-signature genes: Molecular Function*. Heat-map demonstrating the significance of over- and under-represented Panther molecular functions. Red colour signifies an over-representation of genes mapping to a certain term, blue colour an under-representation and white a representation as expected, based on the overall distribution on the array. The colour intensity indicates the negative log of the significance p-value. Numbers presented on the heat map display the percentage of genes within a gene set that map to a certain term. The first column states the overall distribution of a term among the 25,971 genes with detectable expression in the data set, followed by each regional gene set and finally the 'CNS-signature' gene set. FCx, fronto-medial cortex; TCx, temporal cortex; OCx, occipital cortex; HiF, Hippocampus; Str, Striatum; Cb, cerebellum; CNS, 'CNS-signature' genes.

**Figure 6:**
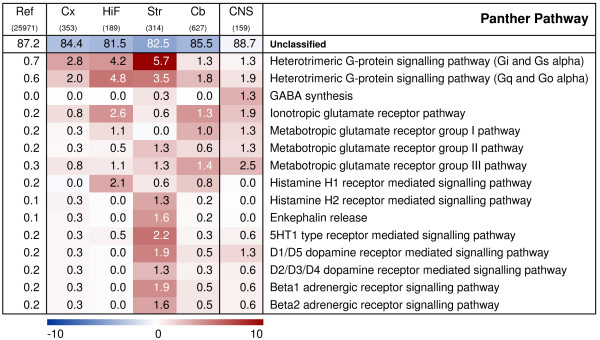
*Functional characterisation of regionally enriched gene sets and CNS-signature genes: Panther Pathways*. Heat-map demonstrating the significance of over- and under-represented Panther pathways. Red colour signifies an over-representation of genes mapping to a certain term, blue colour an under-representation and white a representation as expected, based on the overall distribution on the array. The colour intensity indicates the negative log of the significance p-value. Numbers presented on the heat map display the percentage of genes within a gene set that map to a certain term. The first column states the overall distribution of a term among the 25,971 genes with detectable expression in the data set, followed by each regional gene set and finally the 'CNS-signature' gene set. FCx, fronto-medial cortex; TCx, temporal cortex; OCx, occipital cortex; HiF, Hippocampus; Str, Striatum; Cb, cerebellum; CNS, 'CNS-signature' genes.

The demonstration of distinct gene sets with preference for a certain brain region suggests that the annotations of these genes might reflect functional specialisations of the given regions. Each gene set was therefore mapped to the Panther annotation categories to search for significant over-representations of particular functional groups compared to the overall distribution of the 25,971 genes detected on the AB1700 Rat Genome Survey Array. The most prominent biological processes, molecular functions and pathways over-represented among the regional genes are illustrated in Fig. 4, 5 and 6. The entire lists of over- and under-represented categories are provided as Additional file 2. A substantial fraction of the regional genes were 'unknown' with no functional annotation (as of January 2007). These were unevenly distributed among the regions, comprising 44% (Biological Process) and 45% (Molecular Function) of the cerebellum-enriched genes, but only 35%/38% of the hippocampal genes. In comparison, more than 50% of all the genes represented on the array are still lacking functional annotation.

Certain categories were over-represented in all four regional gene sets, but with a clear regionalisation within the sub-categories. Among the biological processes, 'neuronal activities' were highly represented in all regions, but to a lesser extent in cerebellum than in the other three sites (5% of cerebellar genes vs. ~10% of others; Fig. 4). Signal transduction was also a shared feature, with distinct sub-categories being prominent in different regions. Cell communication was most pronounced in cortex and hippocampus, while G-protein mediated signalling was strong in striatum. Intracellular signalling was highly over-represented in cerebellum and hippocampus; in the latter, calcium-mediated signalling comprised a large proportion of these. It is interesting to note that neurogenesis-related genes, although over-represented to a certain degree in all four brain regions, stood out particularly in hippocampus, constituting almost 10% of the genes with enriched expression, compared to representing 2.2% of the genes present on the array.

This regional specialisation was also reflected among the molecular functions mapped to the enriched gene sets (Fig. 5). Voltage-gated ion channels were generally over-represented, with mainly calcium channels in hippocampus and potassium channels in cortex. Furthermore, two groups of transcription factors were differentially represented. Cerebellum showed a preference for zinc fingers while basic helix-loop-helix transcription factors were heavily over-represented in hippocampus.

Finally, genes mapping to certain signal transduction pathways also gave valuable information on regional specialisation, although only ~20% of the rat genes represented on the array currently has been mapped to a Panther pathway (Fig. 6). Genes involved in heterotrimeric G-protein signalling pathways were over-represented in all four brain regions examined here. Cells of the striatum have a clear preference for the Gi-alpha and Gs-alpha mediated pathways, whereas those of the hippocampus seem to have a slightly higher tendency towards the Gq-alpha and Go-alpha mediated pathways. Both pathways were equally represented in cortex. Thus, the global gene expression data have provided an opportunity to visualise differential use of G-protein mediated signalling pathways in different brain regions.

Another feature of interest is that genes involved in glutamate pathways were over-represented within the enriched gene sets of the cortex, the hippocampus and the cerebellum, but not in the striatum, which instead showed an over-representation of genes involved in dopamine, serotonin and beta adrenergic receptor signalling pathways. In line with this finding, there was also a differential use of glutamate receptors and pathways in cortex, hippocampus and cerebellum. First, genes associated with the ionotropic glutamate receptor pathway were most significantly over-represented in cerebellum, followed by hippocampus and then by cortex. Moreover, within the metabotropic glutamate receptor pathways, members of the group I pathway were preferentially enriched in hippocampus, while the group II and III pathways were almost equally enriched in cortex and cerebellum, group III genes apparently being more numerous than group II genes in both regions.

### 'CNS-signature' genes

Gene expression within the CNS appears to be quite distinct from that of other tissues, both with respect to quantitative (numbers of genes expressed) and qualitative (global expression profiles) measures. It is therefore interesting to study CNS-specific expression, to further understand basic processes that distinguish the brain from other tissues. In order to approach this issue, we aimed at selecting a set of genes showing ubiquitous expression in the CNS and no expression in our non-CNS tissues, thereby constituting 'CNS signature genes' that perform 'CNS-housekeeping' functions necessary to keep the neuronal machinery going.

When a detection threshold of S/N ≥ 3 was applied, we identified 243 genes that were expressed in all CNS samples included in this study but not detected in any of the non-CNS tissues. In order to examine how this particular CNS-specific gene set would change according to the detection level, we increased the S/N-ratio threshold in discrete steps (of size one) from three to fifteen, registering the set of genes at each step. Interestingly, by increasing the detection level from S/N ≥ 3 to S/N ≥ 6, the number of CNS-specific genes increased to a maximum of 337. This number remained quite steady until S/N ≥ 10 (325 genes), after which it decreased gradually (Additional file 3). Of the 243 CNS-specific genes at S/N ≥ 3, only 159 still fulfilled the criterion at S/N ≥ 6 (Additional file 4). The 86 genes that were 'lost' showed marginal expression in some of the CNS regions and were no longer 'detected' in these samples when the threshold was increased. The 180 genes that had been 'gained' at S/N = 6 were ubiquitously expressed within all CNS samples and in addition showed marginal expression outside the CNS. When proposing a CNS gene signature, genes with marked regional differences should probably not be included. Moreover, genes that might have a function both outside as well as within the CNS, such as the 180 genes that are CNS-specific only at higher detection levels, should probably be omitted. The remaining set of 159 genes that were truly CNS-specific and at the same time ubiquitous among all CNS samples, was defined as the "CNS gene signature" in this study and are provided in Additional file 5. The exclusive neuronal distribution of a selection of these genes was confirmed by searching the Symatlas and the GEO databases (data not shown).

In order to support the rationale behind the proposal of the 'CNS signature' gene set, this list was mapped to the Panther annotation categories to search for significant over-representations of particular functional groups (Fig. 3, 4 and 5). The resulting annotations showed a strong neuronal profile. The CNS signature genes are heavily involved in various neuronal activities, especially synaptic transmission. Furthermore, genes involved in ion transport and also cell communication were highly over-represented among the genes. Similar to what was observed for the regionally enriched genes, 'unclassified' genes comprised a significantly smaller proportion (~30%) of the CNS signature genes than would be expected by chance. These previously unknown genes pose as attractive candidates for further study of CNS function.

## Discussions and Conclusion

In the present microarray-based analysis of rat brain, we have studied novel functional relationships between different anatomical regions and their corresponding transcriptomes. We examined three sub-regions of cerebral cortex (fronto-medial, temporal and occipital), hippocampus, striatum and cerebellum, together with three non-CNS tissues, using the Applied Biosystems Array Expression system. In general, our resulting data were highly consistent. Gene expression profiles of replicates were strongly correlated, both with respect to samples from the left and right hemisphere of a given brain region and with respect to samples from the same brain region or tissue in different rats. In comparison, global expression profiles of un-related tissues, such as brain and liver, showed no correlation (Fig. 1).

### Distinct patterns of gene expression in different rat brain regions

According to the calculated correlation coefficients and the unsupervised hierarchical clustering, the transcriptomes of the cortical and hippocampal samples were most similar, followed by the striatal samples, with the cerebellar samples clearly being the most distinct (Fig. 1a and 2). These findings agree well with traditional histological and developmental studies as well as previous expression studies of various brain regions in mice, humans and chimpanzees [4,6-8,10]. The hippocampus and cortex are both forebrain structures and have many cellular and structural features in common, whereas the cerebellum has a distinct developmental origin and a unique cellular composition. Interestingly, the embryonic cellular position along the anterior-posterior axis of the neural tube seems to be to be closely associated with, and possibly a determinant of, the gene expression patterns in adult structures [10].

Within each brain region, expression profiles were highly correlated, irrespective of the hemisphere (left vs. right) and rat from which the sample was derived. We were thus unable to detect systematic differences in the transcriptomes between individual rats as well as between samples originating from left and right hemispheres, indicating that the gene expression programs in a specific anatomical site are highly conserved among individual rats. In humans, the left and right cerebral hemispheres are functionally asymmetric and specialised for distinct cognitive and behavioural functions. A study of human embryonic brains identified a number of transcripts that differed in expression levels between the left and right hemispheres [18]. Moderate asymmetric expression was detected in mouse embryonic cortex as well, although not consistently lateralised to the right or left [18]. The left-right expression differences were however diminished in a 19-week-old human brain [18]. A similar lack of lateralisation in gene expression between adult cortical regions was reported from human and chimpanzee brains, as no genes were found to be differentially expressed between Broca's area located in the left frontal lobe, which is associated with speech production, and the corresponding area in the right hemisphere [4].

The transcriptional profiles of the rat frontomedial, temporal and occipital cortices were highly similar. Although a small number of genes were found to be enriched in either, these were too few to influence the global hierarchical analysis in Fig 2. It has been argued that most genes with differential expression between rat cortical areas may show less than two-fold differences in expression levels, hence cortical differences can be expected to be modest [19]. In contrast, human and chimpanzee cortical regions seem to cluster based on the individual from which they are derived, rather than the inherent cortical region [4]. This effect was observed in both humans and chimpanzees, but was most evident among the human samples, possibly reflecting responses of different individuals to environmental or physiological factors throughout life or immediately before death [4]. Such processes are clearly different in rodents, which have quite restricted individual experiences in their short lives before sacrifice.

Neural tissue has a high molecular complexity, with a large number of different cell types, both neuronal and glial, within a tiny amount of tissue [20]. The laminated structure of the cerebral cortex is particularly heterogeneous and several layer-specific genes have been identified by various molecular approaches [19,21,22]. The sensitivity of microarrays may therefore be suboptimal when profiling complex, heterogeneous tissues such as the cortex. The area-specific molecules, conferring functional specificity, may be expressed in a subset of neurons and are likely to be masked in the presence of other, non-regional, transcripts [19]. Analyses have shown that cellularly complex targets lead to averaged gene expression profiles that lack substantial amounts of cell type specific information [23]. Micro-dissection of individually collected neurons is thus proving to become an important tool in the study of site-specific neuronal expression. A study of GABAergic and glutamatergic neuron populations from various forebrain regions demonstrated that each neuronal subtype has its own transcriptional fingerprint and that the cortical region of origin apparently is of less importance for the global transcriptome in a neuronal population than the functional cell type (i.e. GABA vs. glutamate) [24]. In a similar manner, cortical motor neurons can be distinguished from corresponding somato-sensory projection neurons by increased expression levels of genes that are involved in energy metabolism and protein synthesis [23].

However, considering the four major regions analysed in the present study, it is evident that each major brain region (cortex, hippocampus, striatum and cerebellum, with their combination of neuronal and glial cell types) has its own transcriptional finger-print that distinguishes it from the other regions, indicating that surrounding glial cells also may play a role in conferring regional specificity of neuronal functions.

### Regionally enriched genes reflect patterns of functional specialisations

To our knowledge, no study has been able to decompose specialised functions of distinct brain regions based simply on genes that show enrichment within that region. When we identified and examined such genes, we observed that the annotations of these, with a striking resolution, clearly reflected known functional specialisations of the region in question. In a related study, the resolution was limited to the observation of general neuronal activities in the brain, metabolic processes in the liver and immunological processes in the spleen [1]. Recent reports have showed promising attempts at identifying expression factors, pathways or protein interaction networks that are up-regulated in particular sites of the CNS, but lack our discrimination power, both with respect to gene ontologies and signalling pathways [3,5,7].

A large proportion of the genes significantly enriched in the cortex also showed basic levels of expression in the other brain regions, preferentially hippocampus. This could indicate that general neuronal processes shared by most neural tissues may have a higher activity in the cortex than in other brain regions. In line with this, we observed a strong cortical over-representation of gene products involved in synaptic transmission, cell communication and neurotransmitter release, which agrees well with the extensive synaptic activity in this outermost structure in the CNS that continuously receives, processes and submits information from numerous brain regions and other organs.

On a global scale, the hippocampal transcriptome was closely related to that of cortex. Consequently, many features of gene expression in hippocampus were shared with cortex, resulting in a rather low number of hippocampus-enriched genes. The distinct features of the hippocampus were however immediately obvious from the annotations of these few genes. The role of the hippocampal formation in learning and memory was unmistakably illustrated by a massive over-representation of genes involved in calcium-mediated signalling and neurogenesis, including genes mapping to the ionotropic glutamate receptor pathway and the Gq- and Go-alpha G-protein signalling pathways. As an example, calcium influx through NMDA receptors triggers long term potentiation (LTP) in the post-synaptic cell and this process is involved in the development of synaptic plasticity, which is crucial for learning and memory [25]. These functions also depend on neurogenesis, i.e. proliferation, migration and differentiation of neurons, which has only been established to occur in the olfactory bulb and hippocampus of the adult mammalian brain [26,27].

Striatal and cerebellar genes displayed more region-specific profiles than those of hippocampus and cortex, indicating more specialised functions. The most pronounced effects in the rat striatum included an over-representation of genes linked to dopamine receptor- and G-protein-mediated signalling, as well as the metabolism of cyclic nucleotides. The Gq- and Go-alpha G-protein signalling pathway, involved in adenylyl cyclase activation and inhibition, was particularly over-represented in striatum. These observations are in concordance with two previous analyses [3,7] and illustrate the high neurochemical complexity of striatum and its massive innervation of dopaminergic neurons [28]. In addition, our annotation analysis indicated an exclusive over-representation of myelin proteins in striatum. This is probably a dissection artefact and due to potential contamination from the nearby myelin rich internal capsule.

The genes enriched in the rat cerebellum were somewhat aberrant from those of the other three brain regions by including, to a large extent, genes taking part in active transcription, such as zinc finger- and other transcription factors as well as numerous other genes involved in transcription and nucleic acid metabolism. These categories were more or less under-represented among the genes enriched in the other three brain regions. Similar to what has been observed previously, glutamate receptor signalling components were slightly more numerous among the cerebellar genes [5]. Intriguingly, genes involved in neurogenesis were markedly over-represented among the cerebellar genes, as was observed for the hippocampal genes. Previous studies have concluded that the cerebellum is a site of motor learning [29,30], a mechanism which could be linked to the formation of new neurons or other actions that require neurogenesis-related gene expression. The role of such processes in the cerebellum might not be fully understood at this moment, and studies like ours can be important in revealing new functions of this unique structure.

### Towards a 'CNS gene expression signature'

By hierarchical clustering of samples from all tissues and brain regions included in this analysis, we obtained a complete separation of samples of CNS origin and the remaining tissues and cell lines. Global gene expression profiles of regions within the CNS evidently share a signature that instantly distinguishes these from tissues outside the CNS. This may come as no surprise, in light of many processes that take place exclusively within the CNS. It is nevertheless interesting to investigate this profile further in order to understand the characteristics of CNS-specific operations, as exemplified by our pilot set of 'CNS-signature' genes (see below).

We approached the investigation of the CNS signature by identifying CNS-specific 'housekeeping' genes, i.e. genes showing ubiquitous expression in all CNS regions, but not in the non-CNS tissues. With our restricted set of CNS- and non-CNS samples, the aim was obviously not to define a definite set of genes. Rather, through the annotations of our limited number of 'CNS-signature- genes, to get an indication of which functions and processes CNS-specific 'housekeeping' genes are implicated in. A certain proportion of our genes may show expression outside the CNS, in samples not included here, and also some genes may be absent from yet other CNS regions not included. Still, we demonstrate that the gene set in this study may provide useful new insight into both shared and restricted activities of the CNS.

Among our CNS signature genes, we found numerous genes involved in propagation of the action potential in some way or another. Furthermore, the signature genes comprised various cell adhesion molecules, receptors and signalling molecules. All of these functions are necessary at the synapse to provide sufficient proximity and contact between pre- and post-synaptic neurons, to bind secreted neurotransmitters and to convey longer-term effects of the synaptic activity. The characteristics of our CNS signature genes correlate well with those of a recent, related study, which presented a set of 'pan-CNS-genes', showing highly elevated expression specifically across the CNS compared to non-CNS tissues [7], although the selection of genes was based on other features than in our study.

Interestingly, neurogenesis-related genes are almost as strongly over-represented among the CNS signature genes as among the hippocampal-enriched genes. According to current literature, neurogenesis only occurs in the olfactory bulb and hippocampus in the adult brain [31], although other brain regions, such as the cortex or substantia nigra cannot be excluded [32,33]. Our results from both the regional expression profiling and from this analysis of CNS-specific expression suggest that some of the neurogenesis-annotated genes may have other, ubiquitous functions as well, and that these functions are relevant for their CNS-specific expression patterns. Similar to the regionally enriched gene sets, the CNS-signature genes also had a high over-representation (30%) of signal transduction genes. Indeed, cell communication, including cell adhesion- and ligand-mediated signalling, is apparently shared across several neural regions, whereas there seems to be a regional specificity in the use of cell surface receptor-mediated signalling and intracellular signalling cascades. As mentioned above, G-protein signalling pathways seem to be differentially used between the four brain regions studied here. Accordingly, the CNS-signature gene set only contains three genes mapping to such pathways. GABA-related genes are on the other hand sparsely represented among the regional genes, whereas these are clearly over-represented among the CNS-genes. Thus, analyses such as ours can help the understanding of restricted as well as general processes in the brain and probably also in other tissues and organs.

Novel genes, or 'unknowns', are present both among the regionally enriched genes and the CNS signature genes. In total, we have found around 200 regional genes and 40 CNS genes without any annotation whatsoever except having either restricted or ubiquitous expression in the brain regions studied here and therefore may be important for brain function in some way. We aim at exploring possible functions of these novel genes in future studies.

To conclude, our data indicate that many specialised neuronal functions are present in all CNS regions. It is thus timely to speculate on the degree of redundancy within such shared activities. This topic is highly interesting with respect to the plasticity of the brain and the ability of various regions to gain new functions as replacement processes after brain injury. Further studies should explore the overall characteristics of CNS-specific- versus region-specific gene sets in the brain, as well as examine the functions of numerous annotation-less CNS-enriched genes.

## Methods

### Animals and tissue dissection

Female outbred Sprague-Dawley rats (Mollegaard, Denmark) were housed three per cage under standard 12 hours light/12 hours dark cycle, with food supply limited to 15 g/day and water ad libitum. To avoid stress, the rats were handled by experienced personnel for one month at the animal facility before sacrifice. At 12 weeks of age (bodyweight from 260 to 275 grams) and at middle of light cycle, each rat was quickly anesthetised by isofloruane gas for up to 30 seconds and decapitated in a deep anaesthesia. Skull plates were carefully removed, the brain dissected out and immediately placed in ice-cold RNAlater solution (Ambion, USA) for 3 minutes in order to preserve RNA and allow a more precise dissection. The brain was dissected by a trained neurophysiologist using a binocular dissection microscope on an ice-chilled surface. The following brain regions (with corresponding samples from both right and left side; 15–25 mg tissue per sample) were collected: fronto-medial cortex (FCx), temporal cortex (TCx), occipital cortex (OCx), hippocampal formation (hippocampus; HiF), striatum (Str) and cerebellum (Cb). In addition, liver, kidney and spleen samples (about 100 mg each; crushed/minced) were simultaneously obtained from each animal. Detailed descriptions of dissections are provided in Additional file 6. All samples were put in RNA lysis solution (Applied Biosystems, USA) and frozen at -80°C. The time from initial anaesthesia to the end of the dissection was kept constant around 10 minutes.

### RNA preparation, labelling and microarray hybridisation

10–50 mg tissue from each sample was homogenised using the TissueLyser tissue disruptor (QIAGEN, Germany) for 2× 30 seconds at 20,000 rpm. Total RNA was extracted using the ABI PRISM™ 6100 Nucleic Acid Prep Station (Applied Biosystems, USA). Amount and quality of the extracted RNA was verified by the NanoDrop^® ^ND-1000 spectrophotometer (NanoDrop Technologies, USA) and the Agilent 2100 Bioanalyzer (Agilent Technologies, USA).

All microarray experiments were performed using the Applied Biosystems Expression Array system, which is based upon chemiluminescence detection. One μg of total RNA from each sample was reversely transcribed, amplified and DIG-labelled (DIG-dUTP; Roche, Germany), using the Applied Biosystems Chemiluminescent RT-IVT labelling kit. Amount (10–70 μg) and quality of the DIG-labelled cRNA was controlled by both NanoDrop spectrophotometer and Agilent 2100 Bioanalyzer.

Ten μg of DIG-labelled cRNA was hybridised to the Applied Biosystems Rat Genome Survey Microarray according to the manufacturer's instructions. The AB rat microarray contains 26,857 probes against 27,088 genes, covering 43,508 transcripts, together with about 1,000 control probes. The chemiluminescent signal detection, image acquisition and image analysis of the microarrays were performed on the Applied Biosystems 1700 Chemiluminescent Microarray Analyzer.

### Microarray data analysis

The Applied Biosystems Expression System software was used to extract signals and signal-to-noise ratios (S/N). Only microarrays showing average normalised signal intensity above 5,000 and a median background below 600 were included in the study. Signal intensities were imported into J-Express Pro V2.6 software (MolMine, Norway) [34], where inter-array quantile normalisation was performed in order to minimise the effect of external variables introduced into the data. Quality filtering of unreliable spots (S/N<3) was performed before normalisation. The number of expressed genes in a certain tissue or brain region was determined as the average of the numbers obtained in the biological replicates. Examination of similarities and differences in global gene expression profiles of the various regions, cells and tissues was done by unsupervised, agglomerative hierarchical clustering of mean-normalised data, based on the Pearson correlation coefficient. Correlations between regional gene expression profiles were also calculated using the Pearson correlation coefficient.

Identification of genes that displayed significantly enhanced expression in a certain brain region ("region-specific" or "region-enriched" genes) was carried out by 'Significance Analysis of Microarrays' (SAM) [35]. Unfiltered quantile normalised signal intensities were imported into the TM4 Microarray Software Suite Multi Experiment Viewer 3.1 (TMeV) (TIGR, US) [36]. SAM was performed for each specific tissue against all other CNS and non-CNS tissues. The SAM analysis threshold was set to a highly conservative false discovery rate of zero. In addition, the regional average probe signal intensity was required to be at least two-fold higher than that observed in all other CNS samples.

Functional classification of the SAM-generated lists of genes being preferentially expressed in a certain CNS region (cortex, hippocampus, striatum and cerebellum) and the 'CNS-signature genes' was performed by the Panther Classification System 1.2 [11,37]. Each of the gene lists was compared to the entire list of genes that showed detectable expression in at least one of the 43 samples (25,971 genes, S/N ≥ 3) on the Applied Biosystems Rat Genome Survey Array. Statistically significant over- and under-represented annotation categories were determined by binomial statistics, using the observed number of genes versus the numbers expected by chance within a certain annotation group. Categories with p-values above ~10^-4 ^were rejected; however, some sub-categories provided valuable information on regional specialisations despite not meeting this criterion and were thus included for comparison.

'CNS-signature genes' were found by first identifying CNS-specific genes, i.e. genes not showing detectable expression in any of the non-CNS samples. The signal-to-noise ratio (S/N) was used as the threshold for expression and CNS-specific gene sets were determined for discrete levels of detection, from S/N ≥ 3 to S/N ≥ 15. The set of CNS-specific genes at S/N ≥ 3 may include genes with large regional variations within the CNS, having for example marginal expression in some regions and abundant expression in others. These genes are CNS-specific, but not truly ubiquitously expressed. On the other hand, CNS specific genes at higher S/Ns may include genes, which despite being ubiquitously expressed in the CNS; also show marginal expression outside the CNS, i.e. expression that may be wrongfully discarded at higher S/N thresholds. In order to avoid including such genes in the 'CNS signature', it was required that CNS signature genes were defined as those for which all CNS samples received S/N ≥ 6 and all other samples had S/N<3.

The microarray data are publicly available from ArrayExpress under the accession number [ArrayExpress: E-BASE-4].

## Abbreviations

CNS, central nervous system; FCx, fronto-medial cortex; TCx, temporal cortex; OCx, occipital cortex; HiF, hippocampus; Str, striatum; Cb, cerebellum; S/N, signal-to-noise ratio, SAM, significance analysis of microarrays; FDR, false discovery rate.

## Authors' contributions

CS was responsible for the design of the study, participated in the microarray experiments, analysed the microarray data and drafted the manuscript. AOVM was involved in the design of the study, the tissue dissection and the RNA extraction. RH was involved in the design of the study, was responsible for the RNA labelling and participated in the microarray experiments. HB was involved in the design of the study, the RNA extraction and the microarray experiments. BS was responsible for the tissue dissections and involved in the interpretation of the results. KP was involved in the design of the study and was responsible for establishing the MAGE-ML export pipeline from the local BASE database installation, HAJ was involved in the design of the study, provided the animals and was involved in the tissue dissections. II was involved in the design of the study and gave advice on the data analysis. VMS conceived of the study, was responsible for its design, participated in its coordination and data analysis and helped to draft the manuscript. All authors have read and approved the final manuscript.

## Supplementary Material

Additional file 1 Genes significantly enriched in cortex, hippocampus, striatum and cerebellum, respectively (SAM vs all other samples. FDR = 0. Fold change > 2 vs other CNS samples). Click here for file

Additional file 2 Regional and CNS-signature genes mapping to Panther Biological Processes, Molecular Functions and Pathways, respectively. Click here for file

Additional file 3 The number of CNS-specific genes as a function of the signal-to-noise ratio. The solid line shows CNS-specific genes detected in all CNS samples included in this study, the broken line shows the number of CNS-specific genes detected in at least one of the CNS samples. Click here for file

Additional file 4 'CNS-signature' genes, ubiquitously expressed among all CNS samples and not detected anywhere else. Click here for file

Additional file 5 'CNS-signature' genes, S/N ≥ 6 in all CNS samples, S/N < 3 in all non-CNS samples Click here for file

Additional file 6 Detailed descriptions of dissections of brain regions and tissues. Click here for file

## Figures and Tables

**Table 1:** Average numbers of genes expressed in various brain regions and non-CNS-tissues (S/N ≥ 3)

	**number of genes**	SD*	**percent of total**
**total on array**	26 857		100.0
			
**all Samples**	13 030	± 1 303	48.5
			
**all CNS**	14 408	± 870	53.6
cortex	14 116	± 717	52.6
fronto-medial	13 944	± 490	51.9
temporal	13 826	± 784	51.5
occipital	14 580	± 707	54.3
hippocampus	14 532	± 996	54.1
striatum	14 253	± 909	53.1
cerebellum	14 732	± 1 156	54.9
			
**all non-CNS**	11 389	± 672	42.4
liver	12 014	± 662	44.7
spleen	10 795	± 847	40.2
kidney	11 357	± 321	42.3

*SD, standard deviation

**Table 2:** The ten most significantly enriched genes in cortex, hippocampus, striatum and cerebellum, respectively, compared to all other samples

**AB1700 Probe ID**	**Primary Gene ID^a^**	**Delta score^b^**	**Fold^c^**	**Gene Symbol**	**Gene Name**	**'Validation'^d^**	**Source**
**Cortex**							
20757128	301423	21.2	11.5	LOC501145	similar to KIAA1034-like DNA binding protein	+	Human GeneAtlas
20804233	rCG56789	21.1	4.9	unassigned	unassigned	NA ^e^	No record
22394757	116470	20.9	7.2	Stx1a	syntaxin 1A (brain)	+	Ratlas
22106387	rCG41482	20.6	16.7	unassigned	unassigned	NA	No record
21180100	289440	20.5	5.1	Abhd7 predicted	abhydrolase domain containing 7 (predicted)	NA	No record
21191309	25033	20.1	7.4	Sstr1	somatostatin receptor 1	-	Ratlas
21546223	363102	18.8	5.3	unassigned	unassigned	NA	No record
20813345	312607	18.2	3.9	Pdzrn3 predicted	PDZ domain containing RING finger 3 (predicted)	+	Mouse GeneAtlas
21401322	311078	17.9	5.9	LOC311078	similar to T-Brain-1	+	Mouse GeneAtlas
21849766	rCG63352	17.7	4.9	unassigned	unassigned	NA	No record
							
**Hippocampus**							
21875182	311861	33.3	14.4	RGD1309097 predicted	similar to Hypothetical protein FLJ14810 (predicted)	+	Mouse GeneAtlas
22010557	25153	25.8	9.3	Mas1	MAS1 oncogene	+	Ratlas
22272616	288979	23.3	9.1	LOC288979	similar to complement component 1, q subcomponent-like 2	+	Mouse GeneAtlas
21357331	25672	18.9	5.1	Nr3c2	nuclear receptor subfamily 3, group C, member 2	+	Ratlas
21273900	24785	18.6	10.8	Slc9a4	solute carrier family 9, member 4	-	Ratlas
21372096	79247	17.9	11.2	Htr5b	5-hydroxytryptamine (serotonin) receptor 5B	+	Ratlas
20746536	rCG37048	17.6	6.8	unassigned	unassigned (similar to Potassium channel tetramerisation domain containing 4)	+	Mouse GeneAtlas
21740530	299757	17.3	27.4	Nts predicted	neurotensin (predicted)	-	Mouse GeneAtlas
21310693	54258	14.5	14.0	Il8ra	interleukin 8 receptor, alpha	+	Ratlas
21051913	288207	13.1	5.5	LOC288207	similar to Ephrin type-A receptor 6 precursor (Tyrosine-protein kinase receptor EHK-2)	+	Mouse GeneAtlas
							
**Striatum**							
21655681	78974	46.3	68.1	Six3	sine oculis homeobox homolog 3 (Drosophila)	(+)	Mouse GeneAtlas
22132190	360616	37.4	19.8	Ppp1r1b	protein phosphatase 1, regulatory (inhibitor) subunit 1B	+	Mouse GeneAtlas
21997003	83683	32.8	43.4	Gpr6	G protein-coupled receptor 6	+	Ratlas
21480082	29237	31.7	14.7	Penk-rs	preproenkephalin, related sequence	+	Ratlas
21561963	24318	30.5	50.7	Drd2	dopamine receptor 2	+	Ratlas
21849263	24611	30.3	18.7	Gnal	guanine nucleotide binding protein, alpha stimulating, olfactory type	+	Ratlas
22158395	313445	29.2	8.0	Klhl13	kelch-like 13	+	Mouse GeneAtlas
22195704	24316	29.1	31.4	Drd1a	dopamine receptor 1A	+	Ratlas
22424342	308717	28.8	30.5	Slc35d3 predicted	solute carrier family 35, member D3 (predicted)	+	Mouse GeneAtlas
21577759	29481	28.7	46.7	Rgs9	regulator of G-protein signaling 9	+	Ratlas
							
**Cerebellum**							
22368772	365008	58.5	38.8	LOC361412	similar to ADAMTS18	-	Mouse GeneAtlas
21761660	rCG56756	43.2	202.3	unassigned	unassigned	NA	No record
21883963	498922	38.5	134.3	LOC498922	similar to cerebellin 1 precursor protein	+	Mouse GeneAtlas
22259505	64618	37.8	49.5	Zic1	Zic family member 1 (odd-paired homolog, Drosophila) (HS)	+	Mouse GeneAtlas
21062179	498921	36.6	17.6	LOC498921	LOC498921	NA	No record
22187648	499964	35.9	273.3	LOC499964	similar to engrailed 2 protein	+	Mouse GeneAtlas
21989402	25509	32.6	17.7	Pax6	paired box gene 6 (TS)	+	Ratlas
21979738	287424	31.0	10.0	Aloxe3 predicted	arachidonate lipoxygenase 3 (predicted)	-	Mouse GeneAtlas
21212636	29458	30.0	58.1	Neurod1	neurogenic differentiation 1 (TS)	+	Ratlas
20988804	84012	28.5	51.3	Slc1a6	solute carrier family 1 (high affinity aspartate/glutamate transporter), member 6 (TS)	+	Ratlas

^a ^Celera ID for Celera exclusive genes, otherwise Entrez ID

^b ^Delta score: from SAM analysis (FDR = 0)

^c ^Fold: expression level of gene in a given region relative to the average expression in other CNS tissues.

^d ^Independent 'validation' of the regional enrichment is indicated, showing a '+' if a similar trend is detected by manual inspection in the SymAtlas (Human and Mouse GeneAtlas and Ratlas) [13, 14], otherwise showing a '-'.

^e ^Not applicable, no record of gene in expression database.
