# Supplementary figures and images for: Gene expression profiles in rat brain disclose CNS signature genes and regional patterns of functional specialisation
Source: BMC Genomics. 2007 Apr 4;8:94. doi: 10.1186/1471-2164-8-94 (PMC1853090; doi:10.1186/1471-2164-8-94)

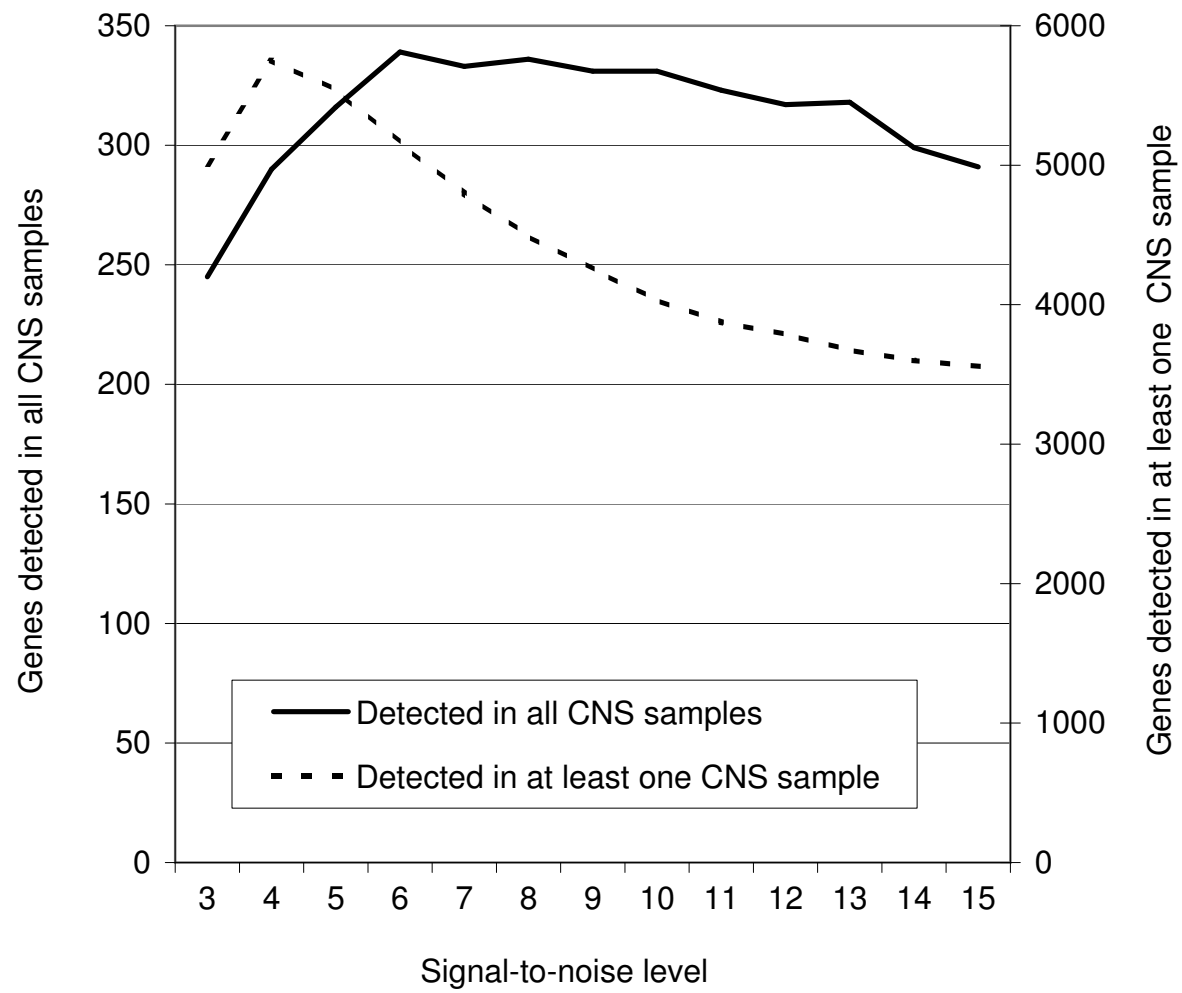

Supplement: Additional file 3 — The number of CNS-specific genes as a function of the signal-to-noise ratio. The solid line shows CNS-specific genes detected in all CNS samples included in this study, the broken line shows the number of CNS-specific genes detected in at least one of the CNS samples. [file 1471-2164-8-94-S3.pdf]
