# Supplementary material for: Gene expression profiles in rat brain disclose CNS signature genes and regional patterns of functional specialisation
Source: BMC Genomics. 2007 Apr 4;8:94. doi: 10.1186/1471-2164-8-94 (PMC1853090; doi:10.1186/1471-2164-8-94)
Supplement: Additional file 4 — 'CNS-signature' genes, ubiquitously expressed among all CNS samples and not detected anywhere else. [file 1471-2164-8-94-S4.pdf]

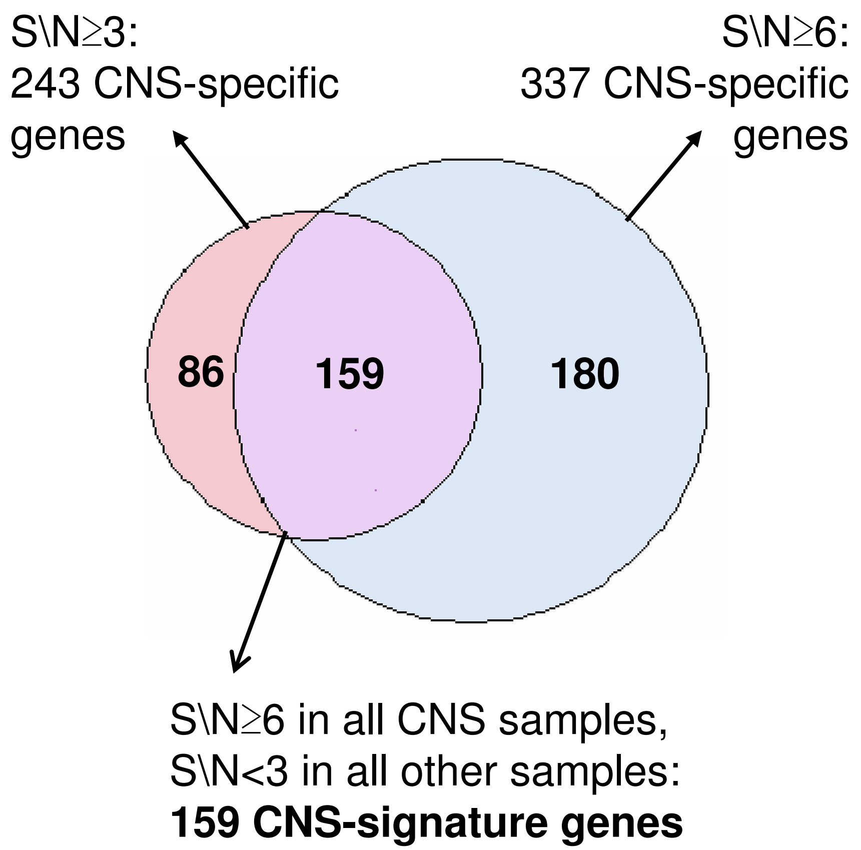

S\N≥3:
243 CNS-specific
genes
S\N≥6:
337 CNS-specific
genes
86
159
180
S\N≥6 in all CNS samples,
S\N<3 in all other samples:
159 CNS-signature genes
